# Hormonal Fluctuations and Premenstrual Exacerbation Across Psychiatric Disorders: Insights for Clinical Practice

**DOI:** 10.1192/j.eurpsy.2026.11953

**Published:** 2026-07-31

**Authors:** T. M. C. Cardoso

**Affiliations:** Psychiatry and Mental Health, Unidade Local de Saúde da Arrábida, Setúbal, Portugal

## Abstract

**Introduction:**

Premenstrual exacerbation (PME) is the cyclical intensification of pre-existing psychiatric symptoms during the luteal phase of the menstrual cycle. PME differs from premenstrual syndrome (PMS) or premenstrual dysphoric disorder (PMDD), as it involves a worsening of baseline psychopathology that does not fully resolve with menstruation. Hormonal fluctuations, especially in estrogen and progesterone, interact with neurotransmitter systems such as serotonin and dopamine. This interaction contributes to mood instability and emotional dysregulation.

**Objectives:**

This narrative review aims to provide an updated overview of PME. It includes definitions, underlying biological and psychosocial mechanisms, and clinical implications across mood, anxiety, psychotic, obsessivecompulsive, personality, and trauma-related disorders.

**Methods:**

A comprehensive narrative literature review was conducted.

**Results:**

Evidence indicates that PME in major depressive disorder is associated with longer episodes, greater anxiety comorbidity, and shorter remission periods, with limited efficacy of standard antidepressant regimens. In bipolar disorder, approximately two-thirds of women report menstrual cycle-related mood fluctuations, and hormonal modulation strategies may offer therapeutic benefit. In psychotic disorders, estrogen appears to play a protective role, and adjunctive hormonal therapies may enhance treatment outcomes. Although research in anxiety, obsessive-compulsive, and trauma-related disorders remains limited, available evidence consistently demonstrates premenstrual symptom exacerbation across diagnostic categories. The underlying mechanisms are multifaceted, with both biological and psychosocial factors contributing to individual susceptibility.

**Conclusions:**

Recognizing PME as a distinct, though overlapping, phenomenon within menstrual-related mood disturbances is essential for improving women’s mental health care. Systematic symptom tracking supports accurate diagnosis and individualized treatment planning. Multimodal approaches—including lifestyle modifications, pharmacological interventions, hormonal therapies, and psychotherapeutic strategies—may improve clinical outcomes and reduce the cyclical burden of psychiatric symptoms.

**Disclosure of Interest:**

None Declared

